# General knowledge norms: Updated and expanded for German

**DOI:** 10.1371/journal.pone.0281305

**Published:** 2023-02-07

**Authors:** Andreas G. Wertgen, Tobias Richter

**Affiliations:** Department of Psychology, University of Würzburg, Würzburg, Germany; Juntendo University, JAPAN

## Abstract

The general knowledge questions introduced by Nelson and Narens (*Journal of Verbal Learning and Verbal Behavior*, *19*(3), 338–368, 1980) have been a valuable research tool in various areas of cognitive research. We translated, updated, and expanded the set of questions for German. We present a total set of 356 general knowledge questions with their recall probability as well as metacognitive measures—confidence and peer judgments—based on a university student sample (*N* = 512). Furthermore, we present response latencies, pairwise correlations between recall probability and metacognitive judgments as well as the most common commission errors. These general knowledge questions can be used in studies with German speaking participants in a broad range of research fields, such as memory, illusory truth, misinformation, and metacognitive processes.

## Introduction

More than four decades ago, Nelson and Narens introduced a set of 300 general knowledge questions with several cognitive and metacognitive measures [1]. Their goal was to provide a normed battery of general knowledge questions covering a wide spectrum of topics (e.g., history, pop culture, science, art, sports, etc.). The question set is based on general information retrieved from fact books, almanacs, trivia books and the like.

Since its introduction, the general knowledge norms have been used frequently in a wide range of research fields, especially in experiments that involve the presentation of general knowledge or violations thereof. For example, research on memory effects [2], metacognition [3], misinformation and fake news [4], validation and text comprehension [5], and illusory truth [6–8] has benefitted greatly from the general knowledge norms. For example, Fazio et al. [6] let participants read correct and false statements to investigate the dependence of illusory truth effects on general knowledge. These statements differed in the likelihood of knowing based on the probability of recall from the general knowledge norms by Nelson and Narens [1].

Even though Nelson and Narens [1] followed the maxim to select their questions and corresponding facts in a way that these would be considered timeless, some of the questions and the associated normative answers are outdated or even incorrect (e.g., “What is the capital of Czechoslovakia?). To this end, Tauber et al. updated and revised the original set of norms three decades later with new samples of university students [9]. More recently, Coane and Umanath [10] compared the performance of older English-speaking adults on general knowledge questions to younger adult performances. Their set of questions was mostly based on the questions first introduced by Nelson and Narens [1] and can be used in developmental research that involves older adults.

Distribution and contents of general knowledge, however, not only vary between different decades and age groups but also between different cultures and countries. Consequently, general knowledge norms created in English and normed with US-American samples cannot be adopted for research purposes in other languages or cultures by simply translating the questions. Based on this consideration, Duñabeitia et al. [11] conducted a norming study based on translated questions of the general knowledge questions from English to Spanish—the first adapted norming study of the general knowledge questions of Nelson and Narens [1] for a different language and culture. To this end, Duñabeitia et al. [11] presented a subset of the original general knowledge norms in Spanish and provided a cross-cultural validation with Tauber et al. [9]. Moreover, Buades-Sitjar et al. [12] presented a new set of 1,364 general knowledge questions normed for a Spanish population with 37 different knowledge fields. To date, the general knowledge norms have not been adapted for German. This may seem surprising considering that the German language is estimated to have about 132 million speakers and is ranked the12th most spoken language in the world, based on *Ethnologue* [13]. Therefore, many psychological studies are based on German-speaking participants.

To fill this gap, we augmented the set by Tauber et al. [9] and constructed a set of general knowledge questions in German. The goal was to provide the general knowledge questions as a reliable research tool for researchers examining German-speaking participants. This goal was approached in two ways. First, we translated the questions into German and tested the translated questions with a German sample of (university) students. However, many of the original questions refer to US-American culture and society, such as historical events, (former) politicians or (old) television shows (e.g., “Andy Griffith was the sheriff of what town on television’s ‘Andy Griffith Show’?”). Hence, we also extended the set with questions that refer to facts that are more typical for German-speaking countries (e.g., “At what age do people in Germany reach the age of majority?”). Otherwise, the approach was similar to Tauber et al. [9]. In particular, we assessed the probability of recall, confidence judgments, peer judgments, and commission errors. Confidence judgments refer to the extent that a participant is confident of a given response (ranging from 0% to 100%). Peer judgments refer to an estimate of the number of people in a hypothetical group of 100 peers that could respond to the presented question correctly. These judgments are useful for researchers to create subsets of questions that vary in these qualities—especially when different judgments vary in their congruency. Thus, we also assessed the correlations between the two types of judgments for each question. Commission errors are the most common incorrect responses for each question. These data permit researchers to provide a more plausible but still incorrect alternative compared with the correct response (for an example, see [14]).

## Method

### Participants

The study included 512 participants (age: *M* = 22.45 years, *SD* = 6.73, range: 18–55 years; 16% men, 83% women) who answered a subset of the translated and expanded general knowledge questions based on Tauber et al. [9]. Participants were recruited through social media and through an online portal for study participation. The study took place in a laboratory (26% of participants) or via an online survey (74% of participants). Most participants were (university) students (91%) and received monetary compensation or study credit. Data were collected in two waves in the laboratory (from November 2018 to December 2018) and in five waves through online participation (first wave: November 2018 to February 2019; second to fifth wave: April 2020 to May 2020).

### Materials

We translated and adapted the 299 general knowledge questions from Tauber et al. [9] which were based on the 300 questions from Nelson and Narens [1] for a German sample. Given that many of the questions used in the surveys of Tauber et al. [9] and Nelson and Narens [1] focus on US-American culture, we added 57 questions that focus on German society and culture (e.g., “What is the vehicle registration number of the city of Hamburg?”) or European context (e.g., “Which state has a blue flag with a yellow cross?”). Some original questions were slightly changed to adapt them for the German sample (e.g., “For which country *was* the Drachma the monetary unit?”). The final set consisted of 356 questions.

### Procedure and measures

Procedures differed slightly between data collection waves. In the two laboratory waves, participants answered a randomized set of questions taken from the whole set of questions for a time limit of 60 min. Participants could answer the open questions at their own speed and instructions were similar to those reported in the study by Tauber et al. [9]. After giving an answer, participants were asked to provide two metacognitive judgments. First, they were asked to judge how confident they were with their answer (in %; confidence judgment) and second, how many of a hypothetical group of one hundred peers would answer this question correct (0–100; peer judgment). These metacognitive judgments had already been used in Tauber et al. [9]. The study was programmed with the experimental software Inquisit [15]. On average, laboratory participants saw 181.68 questions (*SD* = 55.12; range: 63–345) during the 60 min.

The procedure was similar for the online surveys. We could not set a systematic time limit in all online surveys but created four subsets with 89 questions each. A 30 min time limit was imposed in online data collection waves with Inquisit Web. Online participants were assigned to one of the subsets but could participate in all four subsets. Questions were presented in a randomized order. After responding to the general knowledge question, participants were asked to provide confidence and peer judgments—identical to the procedure in the laboratory. Participation in the online surveys was possible with different devices (computer or tablet) and different operating systems (i.e., Linux, Windows, or MacOS). We used the software EFS Survey (Questback Gmbh, Köln, Germany) and Inquisit Web [15] for data collection. Sociodemographic data were assessed for all participants.

We collected response latencies of the world knowledge as a measure of the time a participant took to answer a question. Reliable response latencies were available for data assessed with Inquisit and Inquisit Web. In line with Tauber et al. [9], we only analyzed response latencies of given responses (a total of 37,180 data points). Response latencies longer than 80 seconds were excluded from analysis (489 data points, 0.01% of data). Moreover, response latencies that deviated more than 3 *SD* from the participant or the item mean were excluded from the analysis (1,117 data points, 0.03% of data).

### Ethics statement

Informed consent was obtained before the data collection started. Participants read detailed information regarding ethical guidelines (i.e., the reason for the study, data anonymization and the confidentiality of the data, that participation was voluntary, that they had the right to withdraw their consent to participate at any time, etc.). Only participants who gave their consent could start the study. The study was conducted as part of a research project for which no ethics approval was required from the department. According to German laws and ethical regulations for psychological research (see section 7.3 https://www.dgps.de/die-dgps/aufgaben-und-ziele/berufsethische-richtlinien/#c53;), gathering IRB approval is not necessary if (i) the data are fully anonymized, (ii) the study does not involve deception, (iii) participants’ rights (e.g., voluntary participation, the right to withdraw their data, etc.) are fully preserved, and (iv) participating in the study is unlikely to cause harm, stress, or negative affect. The present study satisfies all of these criteria and thus, no IRB approval had to be obtained.

### Data filtering and scoring

Responses were automatically scored by the experimental software but also scored in a second scoring step by student assistants and the first author. Even though participants were instructed to answer with one word, we accepted correct answers that had more words (e.g., “the holy bible” instead of “bible”). For some questions, an unexpected accurate response was provided (as already indicated by Tauber et al. [9]). In these instances, the accurate response was scored as correct even if we had not anticipated it.

## Results and discussion

The results were collapsed across all waves because the rank correlation between online and laboratory data collection waves indicated a large overlap (Spearman’s ρ = .94, *p* < .001). Note, however, that accuracy was higher in online data collection (probability of correct response: *P* = .41, 95% CI [.41,.41]) compared with laboratory data collection (*P* = .31, 95% CI [.307,.313]), χ*^2^* (1) = 546.78, *p* < .001, φ = .09. On average, confidence judgments for given responses were slightly higher in online data collection (*M* = 67.39, *SE* = 0.71) compared with laboratory data collection (*M* = 61.37, *SE* = 1.02), *t*(509) = 4.51, *p* < .001, *d* = 0.45. A similar pattern occurred in peer judgments with slightly higher judgments for online data collection (*M* = 47.18, *SE* = 0.51) compared with laboratory data collection, (*M* = 44.54, *SE* = 0.83), *t*(510) = 2.65, *p* = .008, *d* = 0.27. For figures showing the frequencies of the probability of recall, confidence and peer judgments split between online and laboratory surveys, please see the supplementary material (S1–S6 Figs).

**Fig 1:**
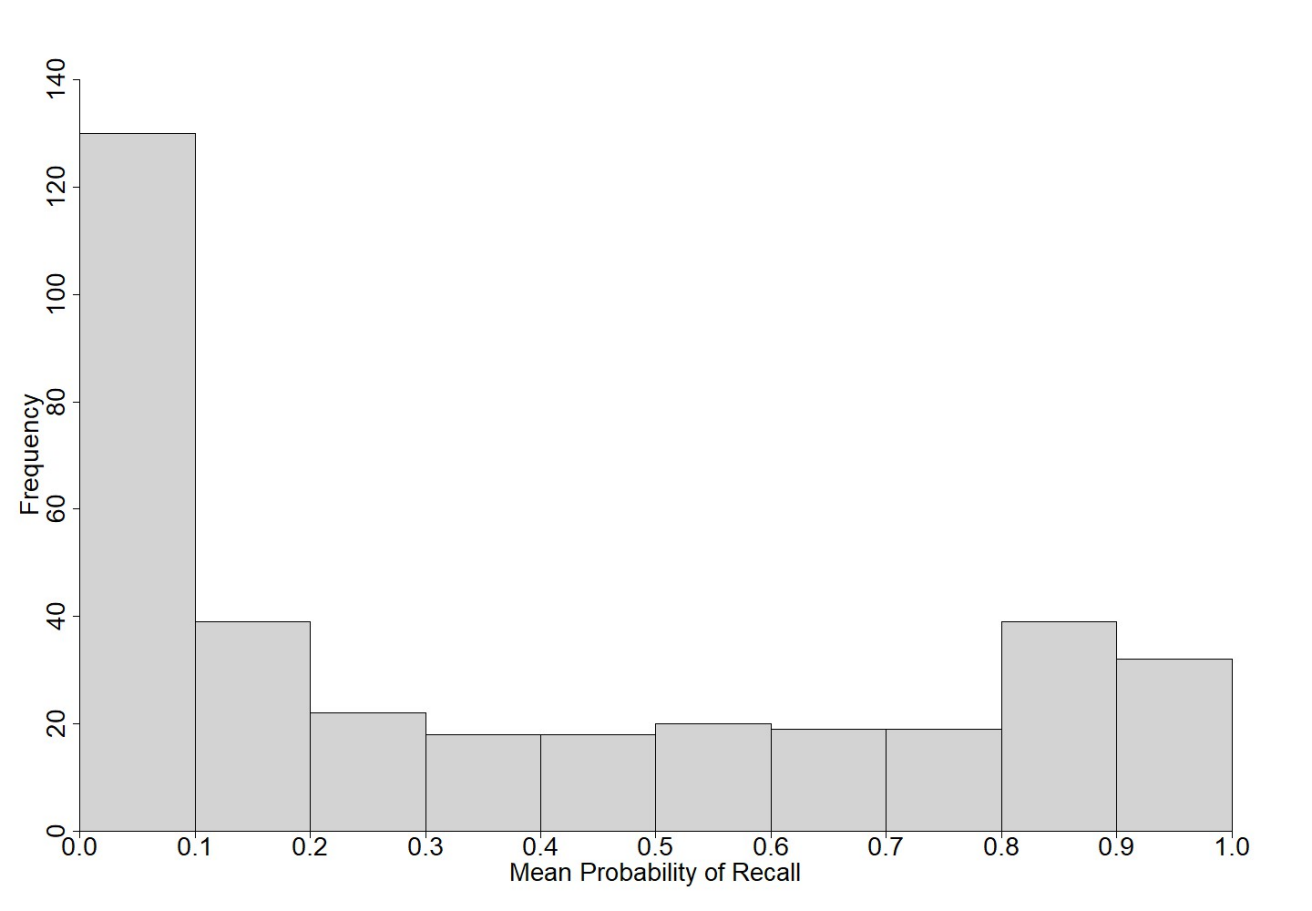
Histogram of the mean probability of recall for all questions.

Participants answered a total of 72,277 questions with 26,639 scored as correct (37% correct). Out of the 45,638 incorrect responses, almost 36% were commission errors. On average, participants answered 142 questions (*SD* = 74) with a range of 17 to 356 questions. Fig 1 shows the frequency of the probability of recall. Participants gave an average of 81 (*SD* = 45) confidence judgments (range: 5–282; Fig 2) and 140 (*SD* = 73) peer judgments (range: 16–356; Fig 3). Comparable to Tauber et al. [9], the total number of responses per questions ranged from 171 to 225 (*M* = 203, *SD* = 10). The results for each question are displayed in S1 Table. Questions are ordered by rank, starting with the highest probability of correct recall. The rank order based on Tauber et al.’s [9] U.S. sample is presented for comparison for the 299 questions used in both studies. S1 Table also provides the probability of recall, the number of correct and incorrect responses, response latencies (in seconds), proportion of error type (commission vs. omission), probability of error, mean confidence judgments, mean peer judgments and the correlation between confidence judgments, peer judgments, and probability of recall. Additionally, S1 Table presents the results of significance tests for comparisons between the present probabilities of correct recall and those reported in Tauber et al. [9]. S2 Table provides details about the most frequent commission errors with absolute and relative frequencies. S1 and S2 Tables can be found in the supporting information.

**Fig 2:**
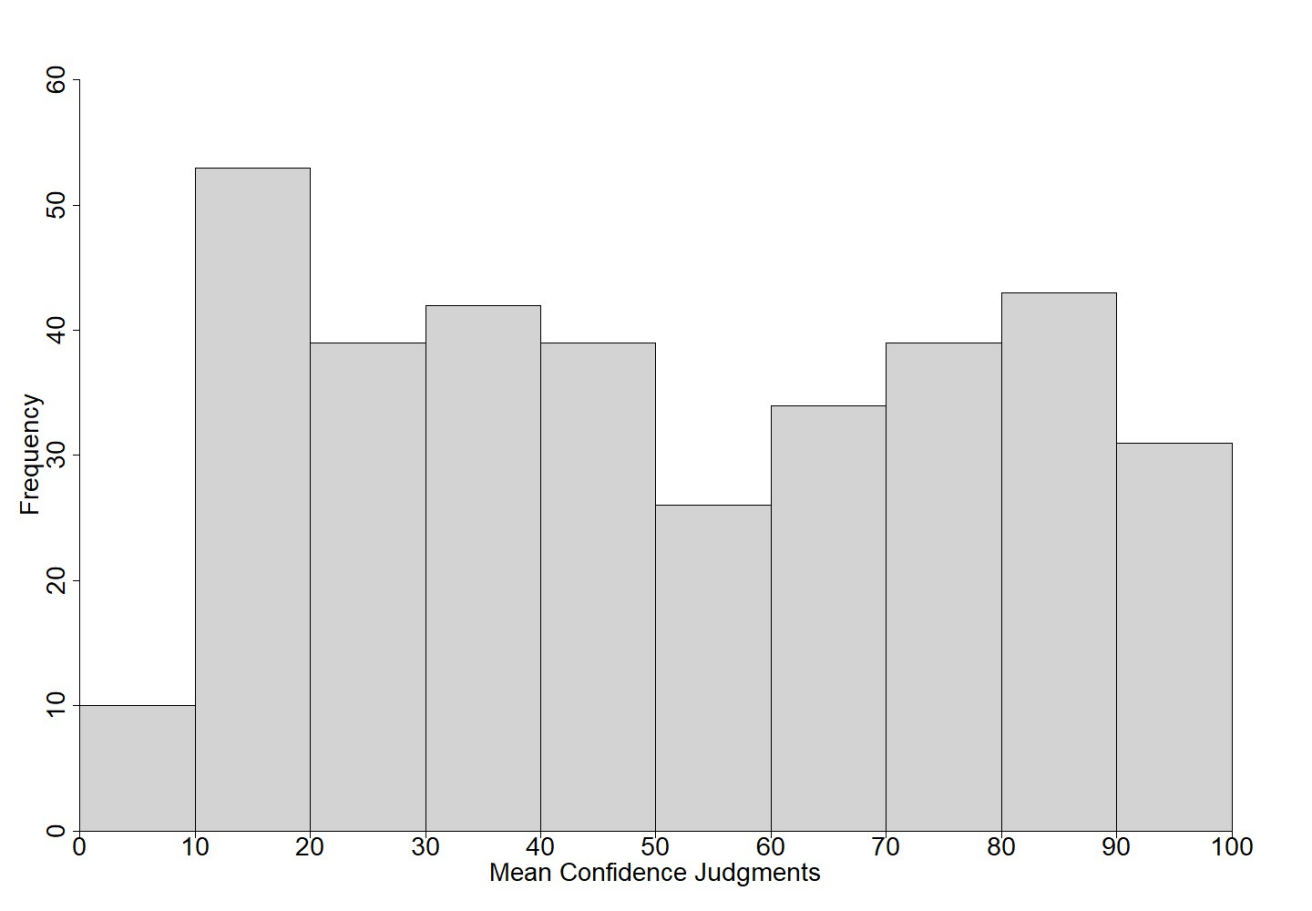
Histogram of the mean confidence judgments for all questions.

**Fig 3:**
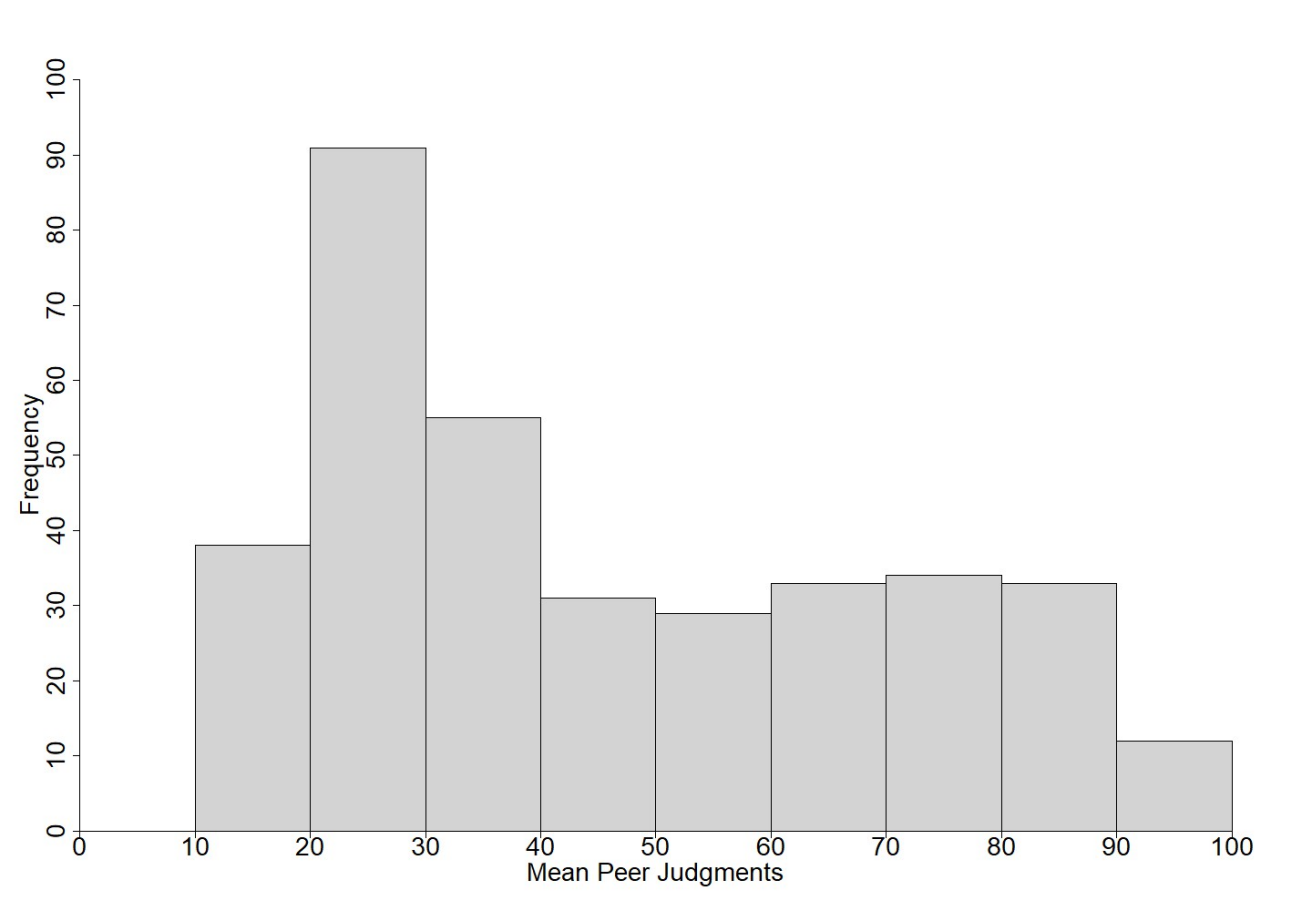
Histogram of the mean peer judgments for all questions.

In this study, we presented an update and extension of the general knowledge norms first introduced by Nelson and Narens [1]. We expanded the existing 299 questions with more typical German and European questions. Performance for questions tailored to the German or European context was better compared to the original questions. Twenty of the 33 questions (60.6%) with a probability of recall ≥ .90 were new questions (i.e., typical German). About 39% of the knowledge questions received almost no correct answers, with a probability of recall < .10. These questions were all from the original set of questions [1,9].

The questions are relatively difficult because of the cued-recall format [10,16], especially for knowledge that is stored in memory but not easily accessible, such as marginal knowledge, which is more likely to be detectable by multiple-choice questions [16]. If needed, our set of general knowledge questions can be transferred easily into multiple-choice questions. Distracters could be constructed based on the most frequent commission errors (S2 Table). These answers represent incorrect but plausible alternatives.

**Fig 4:**
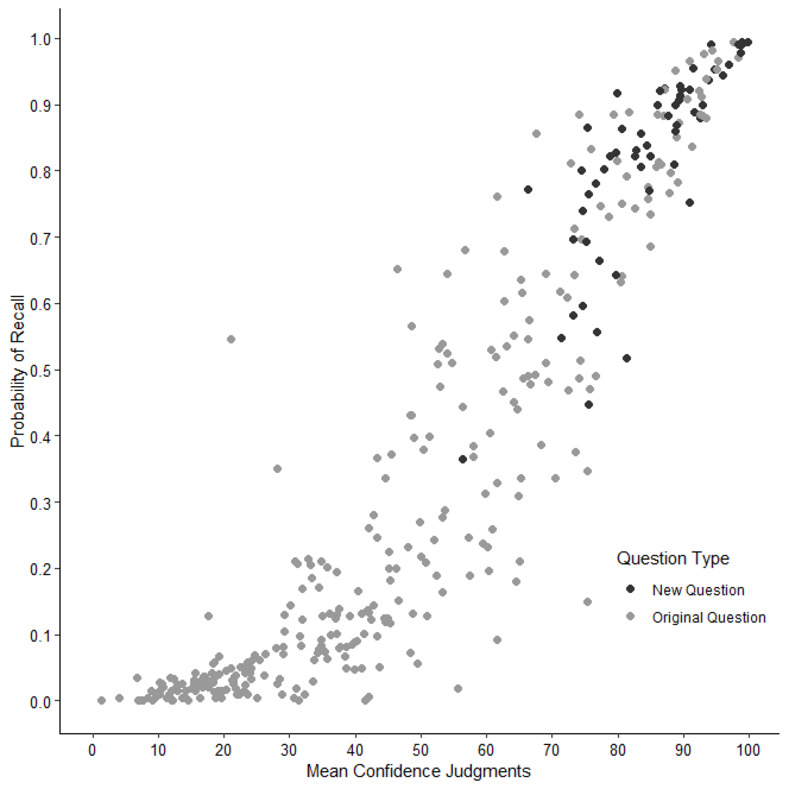
Association between probability of recall and mean confidence judgment per question. Each dot represents one question. Confidence judgments were only analyzed for given responses. Higher mean confidence judgments per question were associated with higher probability of recall.

**Fig 5:**
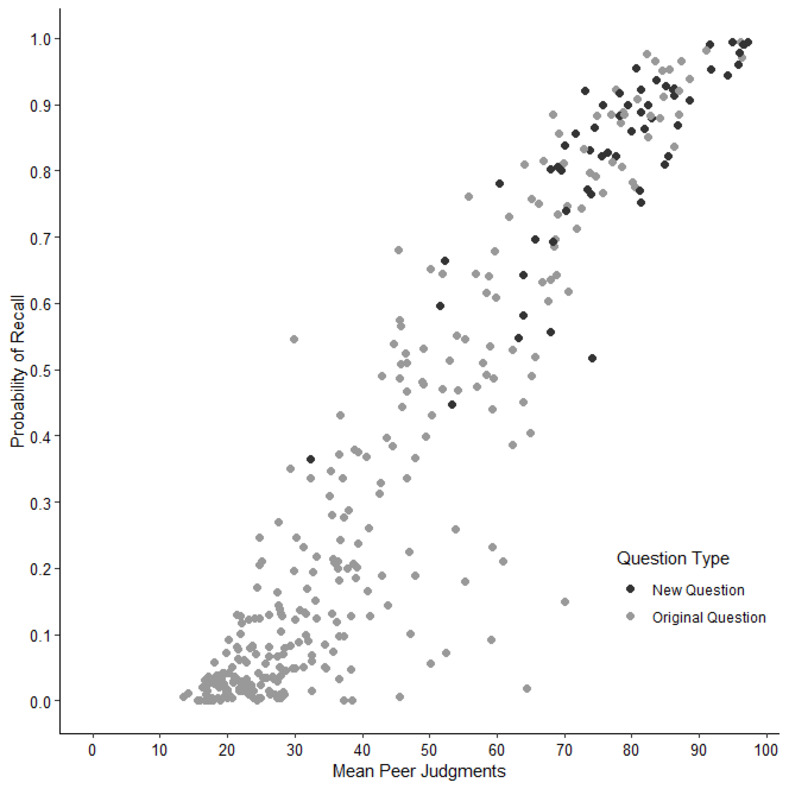
Association between probability of recall and mean peer judgments per question. Each dot represents one question. Higher mean peer judgments per question were associated with higher probability of recall.

Participants judged their confidence as higher with increasing probability of correct recall of the general knowledge questions (*r* = .93, *p* < .001; Fig 4). Similarly, correct recall was strongly and positively related to the estimated percentage of hypothetical peers that could answer the questions correctly (*r* = .94, *p* < .001; Fig 5). Interestingly, participants were fairly confident with some of their incorrect responses indicating a high applicability for psychological research based on these commission errors (e.g., as plausible alternatives).

**Fig 6:**
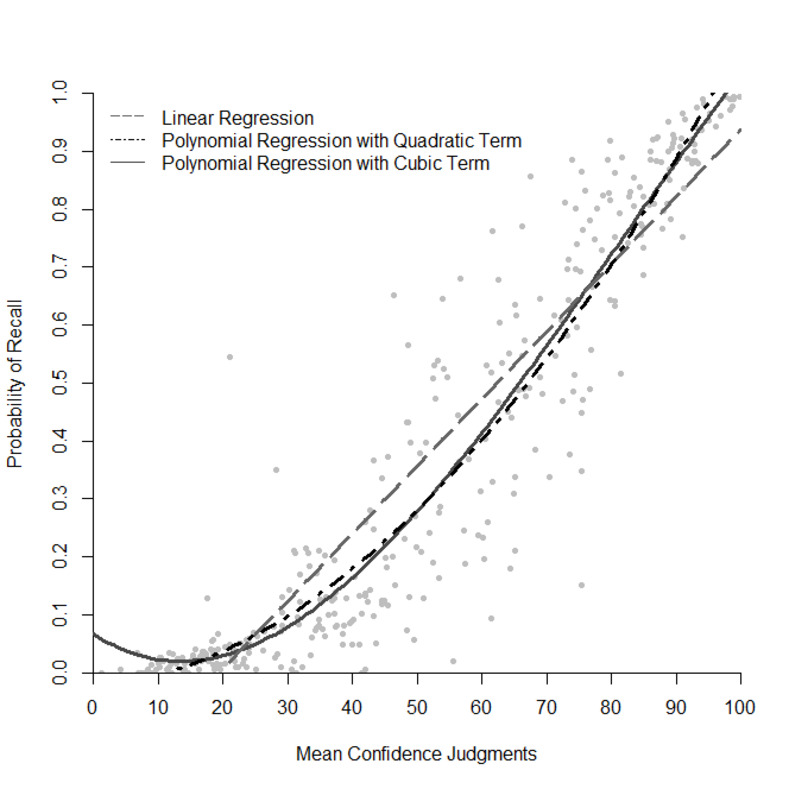
Relationship between the mean confidence judgments per question and the probability of recall modelled with linear and polynomial regression. Each dot represents one question. Confidence judgments were only analyzed for given responses. Regression models are hierarchical.

**Fig 7:**
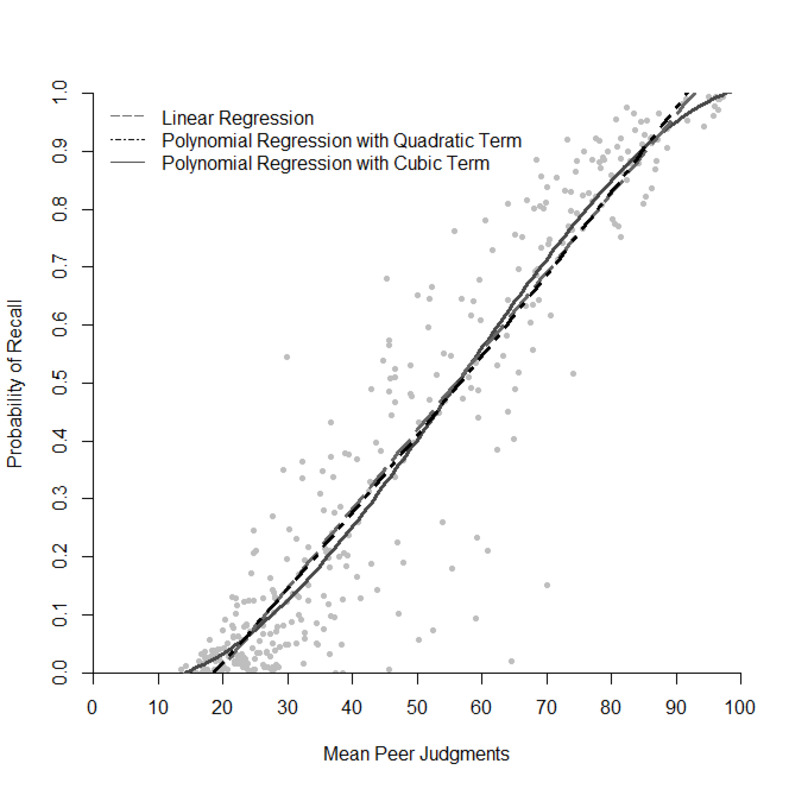
Relationship between the mean peer judgments per question and the probability of recall modelled with linear and polynomial regression. Each dot represents one question. Regression models are hierarchical.

Moreover, we analyzed the relationship between the metacognitive judgments and the probability of recall with exploratory regression analyses. Figs 4 and 5 indicate that the relationship between the metacognitive judgments and the probability of recall may be nonlinear. To this end, we compared linear and polynomial regressions up to the third degree (Figs 6 and 7) [17]. In all regression models, the confidence or peer judgment were entered as a predictor for the probability of recall. Analyses were performed on the question level, that is, probability of recall, peer and confidence judgments were averaged across participants for each question.

Table 1 provides the estimates and significance tests of the effect on the confidence judgements for the probability of recall for each question. The goodness of fit between the three models varied slightly but a regression model with a cubic term (Model 3) yielded the best fit with an adjusted *R*^2^ = .898. This indicates that the relationship between the confidence judgments and the probability of recall is nonlinear. In sum, confidence judgments significantly predicted the probability of recall, with higher confidence judgments leading to a higher probability of recall. However, low confidence judgments (< 20) were relatively weak in differentiating the probability of recall, while for higher confidence judgments the increase in confidence aligned with an increase in the probability of recall. One possible explanation may be that some questions tricked participants to be slightly overconfident in their response. However, the nonlinear pattern may also be due to the scaling of the focal variable: Probability of recall is bounded at 0 and 1, restricting the variance of values approaching 0 and 1 and leading to a “flattening” of the curve for very low and very high values of the predictor [17, p. 240].

**Table 1:** Regression estimates and model comparisons of mean confidence judgments on the probability of recall.

Variable	Model 1				Model 2				Model 3			
	Est.	*SE*	*t*	*p*	Est.	*SE*	*t*	*p*	Est.	*SE*	*t*	*p*
(Intercept)	-22.58	1.44	-15.73	< .001	-2.39	2.27	-1.05	.293	6.61	3.75	1.77	.078
Linear Confidence Judgment	1.16	0.02	47.06	< .001	0.10	0.10	1.00	.318	-0.71	0.29	-2.46	.015
Quadratic Confidence Judgment					0.01	< 0.01	10.67	< .001	0.03	0.01	4.60	< .001
Cubic Confidence Judgment									< -0.01	< 0.01	-3.00	.003
Hierarchical Model												
Model	*R^2^*	*F*	*df*	*p*	Δ*R^2^*	*F*	*df*	*p*				
Model 1 (Linear)	.862	2214	1, 354	< .001	.862	2214.4	1, 354	< .001				
Model 2 (Quadratic)	.895	1517	2, 353	< .001	.033	113.76	1, 353	< .001				
Model 3 (Cubic)	.898	1037	3, 353	< .001	.002	9.00	1, 352	< .001				

*Note*. The probability of recall was transformed to a scale from 0 to 100 for a simplified interpretation of the coefficients. The transformed scale matches the scale of the mean confidence judgments.

Table 2 provides the estimates and significance tests of the effect of the peer judgments on the probability of recall for each question. Again, the goodness of fit between the three models varied slightly. The best fit (adj. *R*^2^ = .888) was achieved by accounting for the nonlinearity with including a cubic term (Model 3) indicating that the relationship of peer judgments and the probability of recall is nonlinear. Peer judgments significantly predicted the probability of recall, with higher peer judgments leading to a higher probability of recall with a weaker association close to the endpoints of the peer judgements. Interestingly, there was a mismatch between peer judgments and the probability of recall for very low probabilities of recall. Participants seemed to judge their hypothetical peers to be more successful in correctly answering questions that, in fact, had a probability of recall close to 0. Similarly, participants seemed to overestimate their peers for questions with a probability of recall close to 1. Again, a likely explanation for this pattern is that the scale of probability of recall is bounded at 0 and 1.

**Table 2:** Regression estimates and model comparisons of mean peer judgments on the probability of recall.

Variable	Model 1				Model 2				Model 3			
	Est.	*SE*	*t*	*p*	Est.	*SE*	*t*	*p*	Est.	*SE*	*t*	*p*
(Intercept)	-26.23	1.36	-19.24	< .001	-22.93	3.21	-7.14	< .001	1.00	7.11	0.14	.888
Linear Peer Judgment	1.36	0.03	52.06	< .001	1.20	0.15	8.26	< .001	-0.56	0.49	-1.15	.252
Quadratic Peer Judgment					< 0.01	< 0.01	1.13	.258	0.04	0.01	3.88	< .001
Cubic Peer Judgment									< -0.01	< 0.01	-3.76	< .001
Hierarchical Model												
Model	*R^2^*	*F*	*df*	*p*	Δ*R^2^*	*F*	*df*	*p*				
Model 1 (Linear)	.884	2710	1, 354	< .001	.884	2710.10	1, 354	< .001				
Model 2 (Quadratic)	.884	1357	2, 353	< .001	< .001	1.29	1, 353	.258				
Model 3 (Cubic)	.888	942.90	3, 352	< .001	.004	14.13	1, 352	< .001				

*Note*. The probability of recall was transformed to a scale from 0 to 100 for a simplified interpretation of the coefficients. The transformed scale matches the scale of the mean peer judgments.

Considerable differences were found between the question ranks in the present study and the ranks presented by Tauber et al. [9]. For example, the question with the highest probability of recall obtained with the U.S. sample in 2012 (“What is the name of the horse-like animal with black and white stripes?”) was only ranked 10th in 2020 (or 4th when only the original questions are considered). The rank correlation (Spearman’s ρ) between 2012 [9] and 2020 (our data) was .69 (*p* < .001). Thus, the general knowledge norms converged to some extent, but this convergence was far from perfect, a pattern that seems plausible given the differences in the linguistic and cultural contexts and the time span of eight years between Tauber et al. [9] and this study.

The general knowledge norms used in the present study must be interpreted in light of its limitations. The differences in results of Nelson and Narens [1] compared with Tauber et al. [9]—and to some extent also our results compared with Tauber et al.—indicate that general knowledge norms change over time. Hence, some of the general knowledge norms reported in this study are likely to be accurate for only a certain time period. Further norming studies in the near future are needed to preserve suitable norms. Moreover, we deem additions to the general knowledge questions to be quite useful for research with German-speaking participants, but the norms might not be generalizable to different European countries or languages. Our sample mostly consisted of university students with a high education. More than 97% had received Abitur (graduation from an academic-track high school), which indicates a typical selection effect found in most published psychological experiments with German populations. However, the norms cannot be extrapolated to the German society at large or specific age groups.

The probability of recall was higher in online data collection compared with data collection in laboratories. One possible explanation is that cheating occurred in the online data collection. However, for various reasons, this explanation seems unlikely. First, offline participants mostly consisted of psychology students, whereas online participants were mostly teacher students from a broad range of academic disciplines (e.g., literature, mathematics, geography). Thus, the higher probability of recall in online vs. offline participants might reflect diverse expertise rather than non-compliance with the instructions. Second, the experimental software covered the whole screen with no easy means for switching to a web browser or search engine. Third, the participant with the highest accuracy only reached an accuracy of .84 (95% CI [.72,.96]), which seems low for cheating. Nevertheless, some concern persists. Of course, only laboratory data collection ensures strict control of participant behavior during the study.

Similarly, the mean confidence and peer judgments were higher in online data collection compared with collection in laboratories. Several aspects of the data collection modes can be considered when discussing the differences found with peer and confidence judgments [18]. First, it seems plausible that higher confidence and peer judgments accompany higher probability of recall, as the likelihood for a correct answer and the confidence in that answer as well as the confidence in the answers of hypothetical peers should be related. Thus, the differences in the judgments may reflect the differences in the probability of recall between data collection modes. Second and further to that point, participants in the online data collection saw less questions and the duration was half as long as in the laboratory data collection. Hence, online participants may have sustained a higher level of on-task focus and motivation to comply while answering the questions resulting in better performance and higher ensuing judgments. Third, the data collection mode may exert an influence on participants. While strict control of participant behavior can only be ensured by laboratory data collection, it may induce specific contextual effects on participants. For instance, the presence of an experimenter may induce a feeling that the participant might be tested, especially when some questions target relatively unknown facts. This somewhat discomforting feeling may result in less confidence and may reduce the corresponding judgments compared with a data collection in a familiar setting without an experimenter and other participants present.

To conclude, despite these limitations, the questions and corresponding facts of the general knowledge norms can be used in multiple research endeavors, for example, in experimental conditions that are designed to control for prior knowledge. The knowledge questions span a wide range of difficulty (probability of recall ranging from .000 to .995). Therefore, the knowledge questions and the corresponding responses can be used in studies with German-speaking participants to design experimental questions with even fine-grained variations of probability of recall or for creating German stimulus material based on general knowledge.

## Supporting information

S1 Fig Histogram of the mean probability of recall for all questions assessed with online surveys. (TIFF)

S2 Fig Histogram of the mean probability of recall for all questions assessed with laboratory surveys. (TIFF)

S3 Fig Histogram of the mean confidence judgments for all questions assessed with online surveys. (TIFF)

S4 Fig Histogram of the mean confidence judgments for all questions assessed with laboratory surveys. (TIFF)

S5 Fig Histogram of the mean peer judgments for all questions assessed with online surveys. (TIFF)

S6 Fig Histogram of the mean peer judgments for all questions assessed with laboratory surveys. (TIFF)

S1 Table Probabilities of recall (with confidence intervals), mean response latencies, proportions of errors (with confidence intervals), confidence judgments (with confidence intervals), peer judgements (with confidence intervals), and correlations between the judgements and the probability of recall for 356 general world knowledge questions. (XLSX)

S2 Table Overview of the most frequent commission errors for questions with a proportion of commission errors of .05 or greater of the total responses. (XLSX)
