# Supplementary figures and images for: General knowledge norms: Updated and expanded for German
Source: PLoS One. 2023 Feb 7;18(2):e0281305. doi: 10.1371/journal.pone.0281305 (PMC9904464; doi:10.1371/journal.pone.0281305)

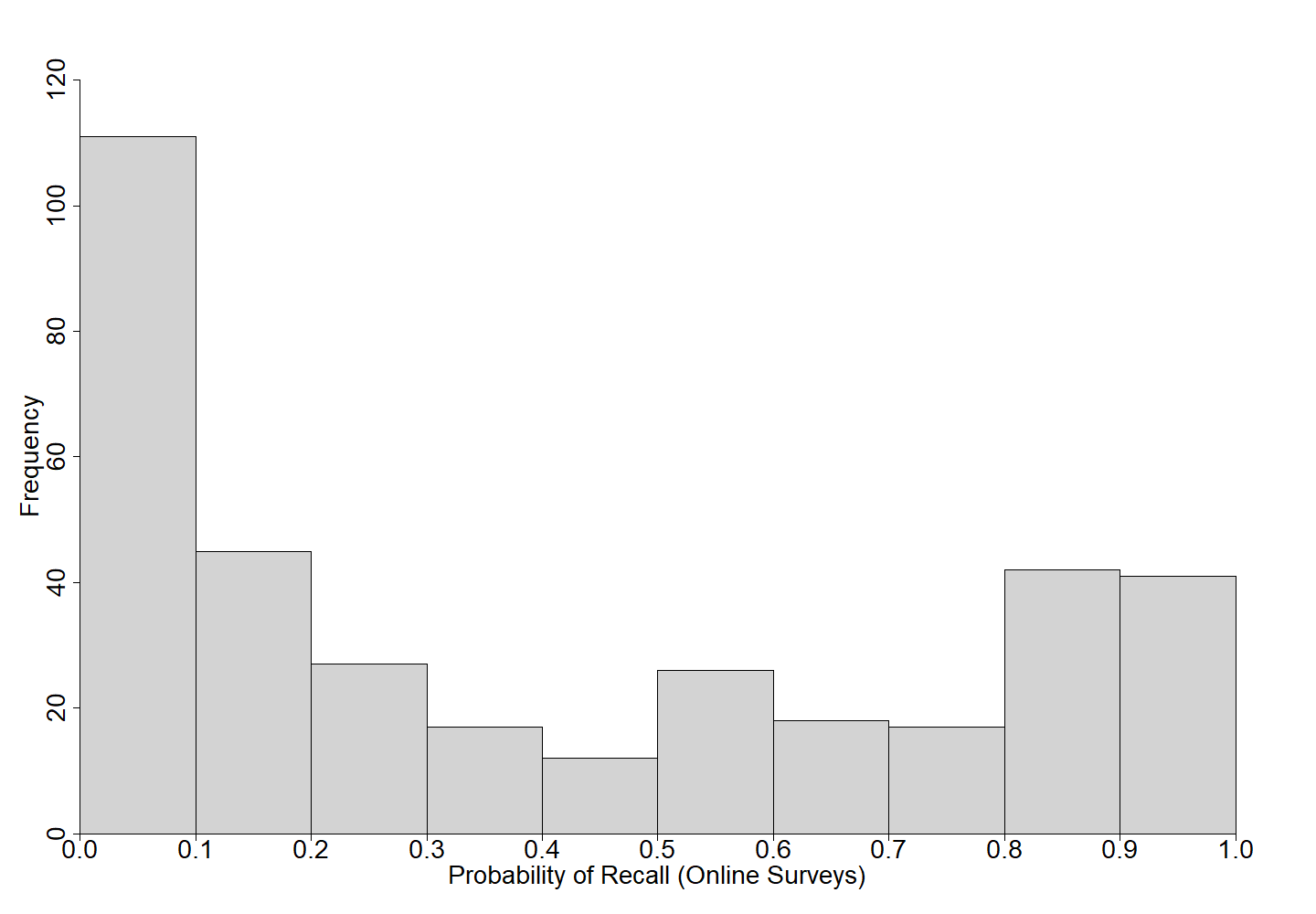

Supplement: S1 Fig — (TIFF) [file pone.0281305.s001.tiff]

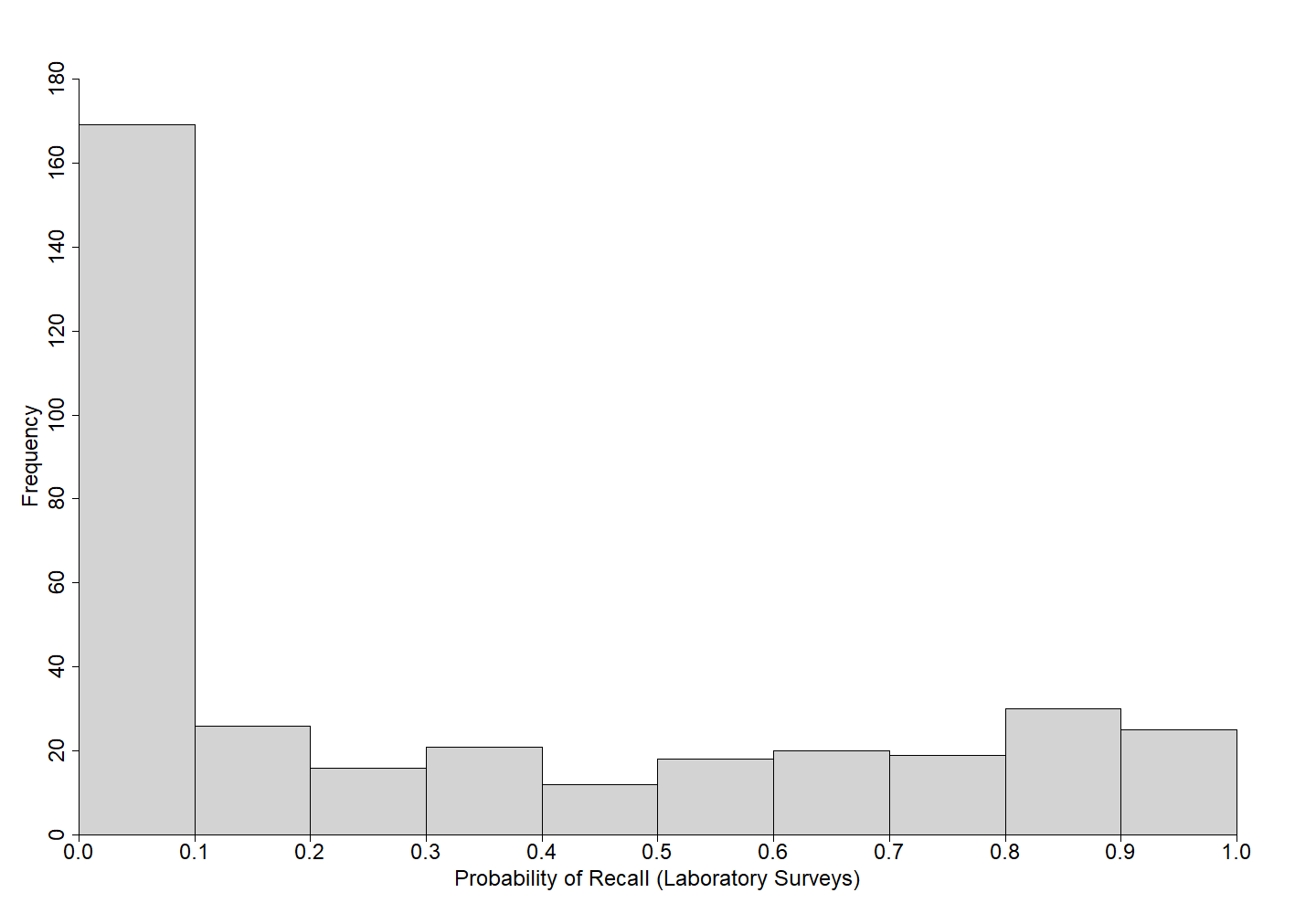

Supplement: S2 Fig — (TIFF) [file pone.0281305.s002.tiff]

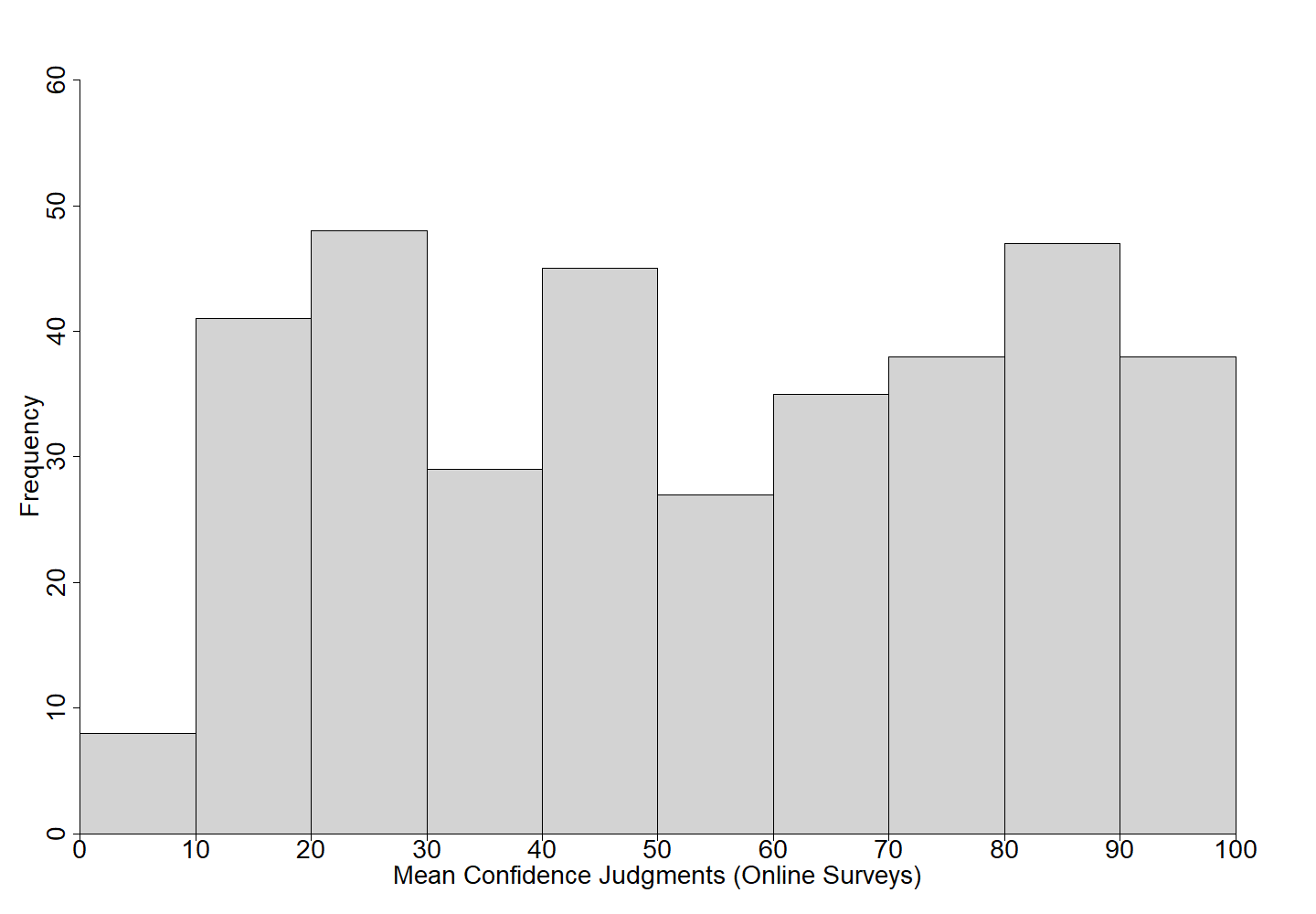

Supplement: S3 Fig — (TIFF) [file pone.0281305.s003.tiff]

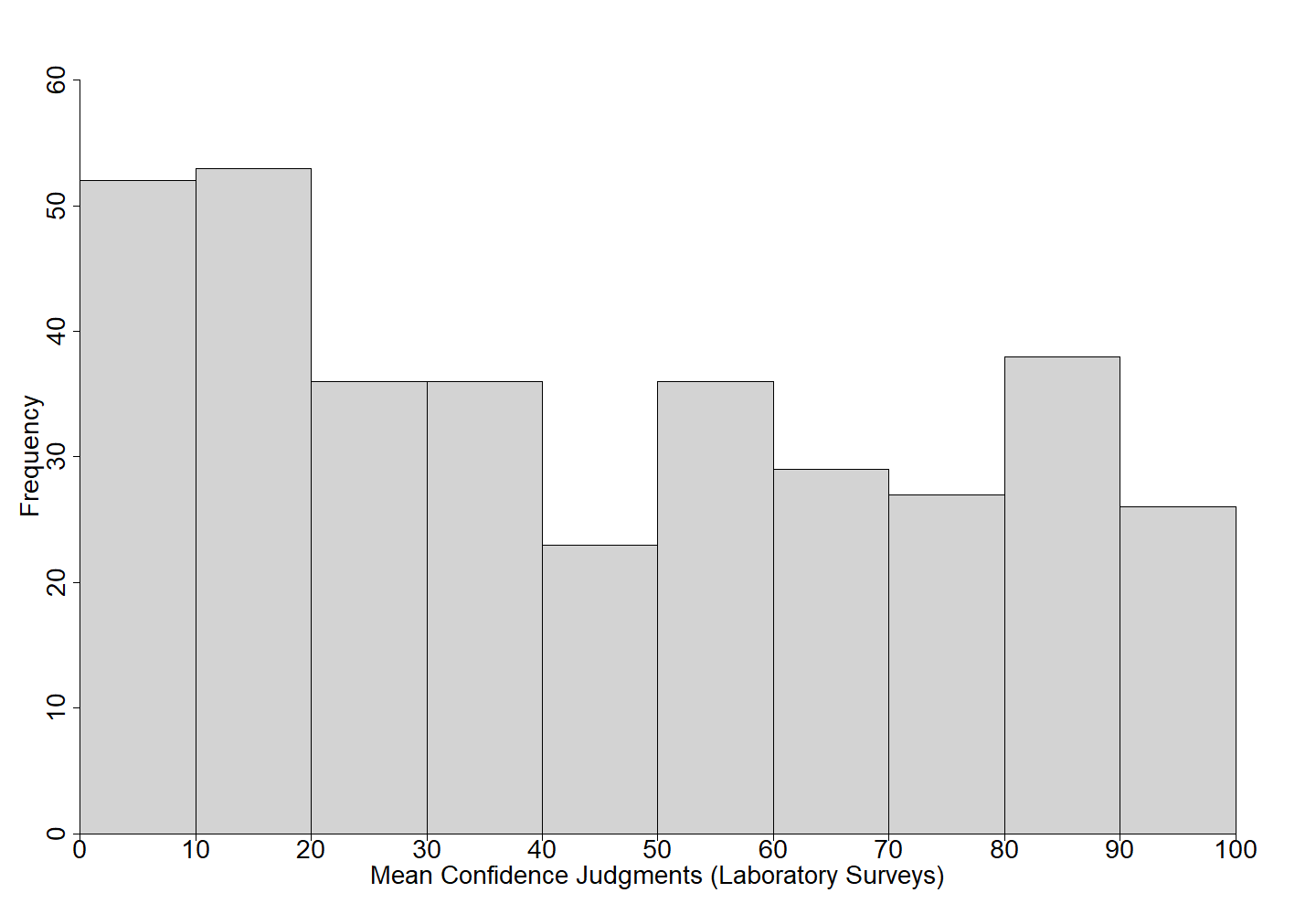

Supplement: S4 Fig — (TIFF) [file pone.0281305.s004.tiff]

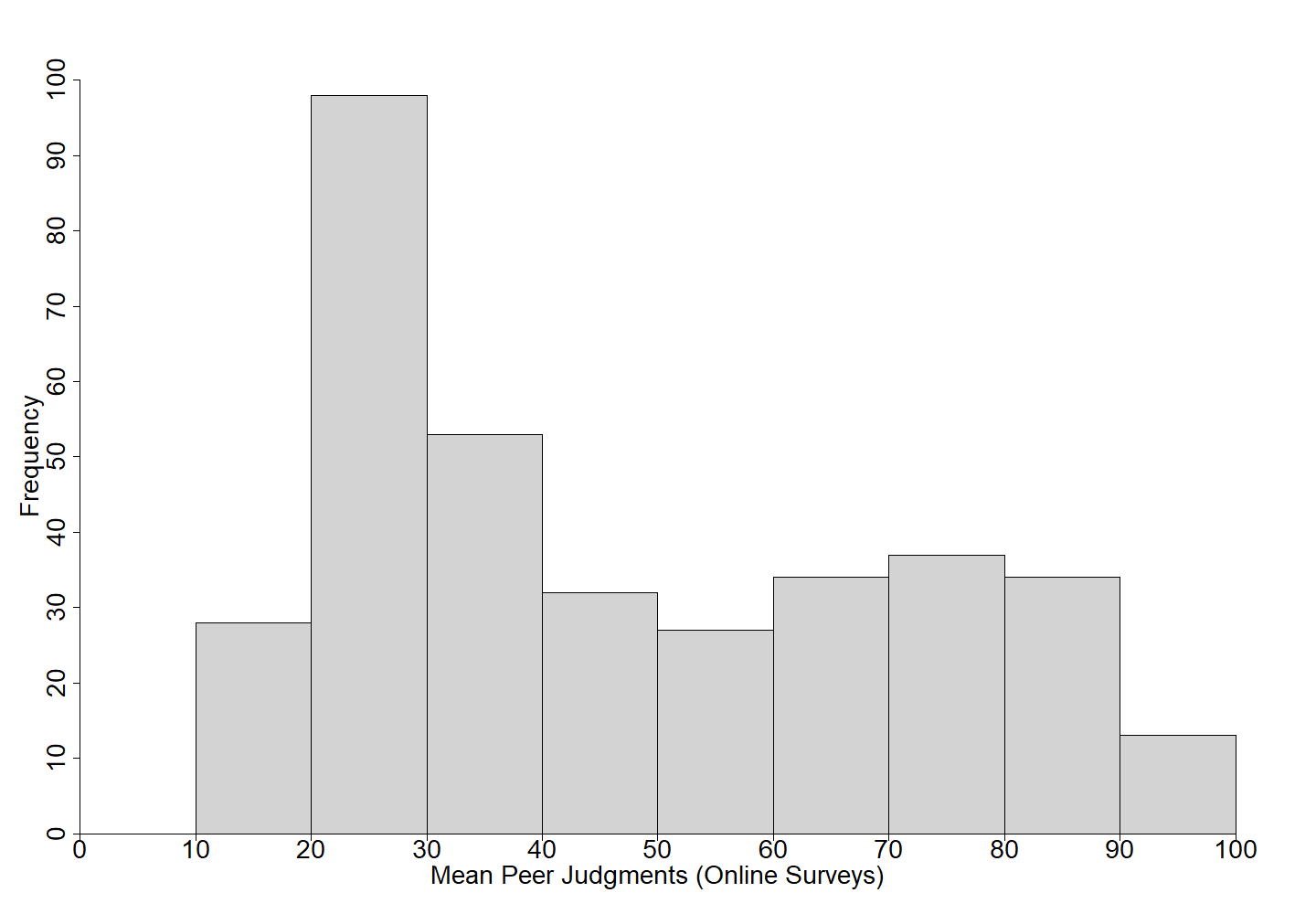

Supplement: S5 Fig — (TIFF) [file pone.0281305.s005.tiff]

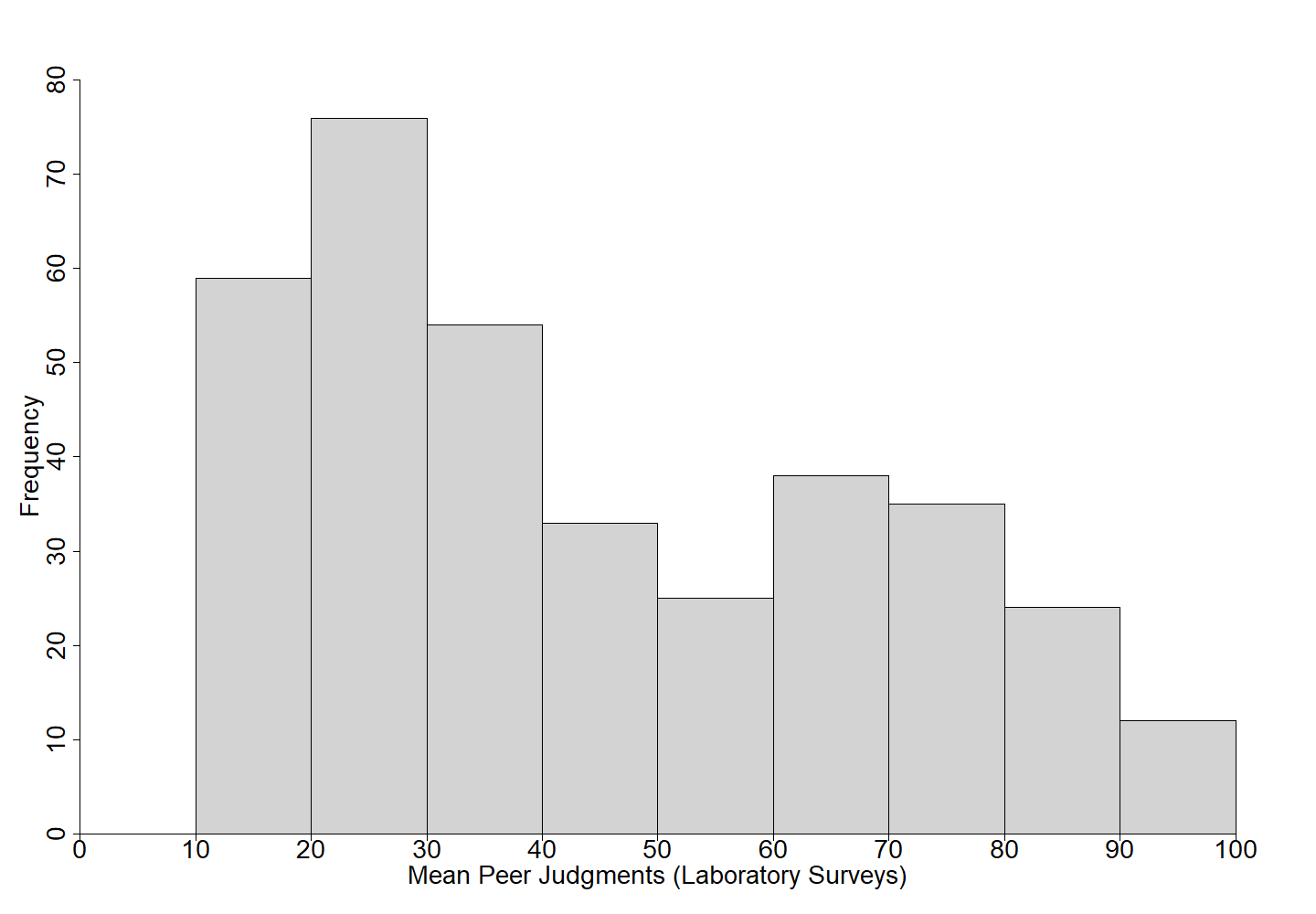

Supplement: S6 Fig — (TIFF) [file pone.0281305.s006.tiff]
